# Replicated association of the single nucleotide polymorphism in *EDG1 *with marbling in three general populations of Japanese Black beef cattle

**DOI:** 10.1186/1756-0500-3-66

**Published:** 2010-03-11

**Authors:** Shin Sukegawa, Takeshi Miyake, Yoichi Takahagi, Hiroshi Murakami, Fumiki Morimatsu, Takahisa Yamada, Yoshiyuki Sasaki

**Affiliations:** 1 Research and Development Center, Nippon Meat Packers, Inc, Tsukuba, Ibaraki 300-2646, Japan; 2 Laboratory of Animal Breeding and Genetics, Graduate School of Agriculture, Kyoto University, Sakyo-ku, Kyoto 606-8502, Japan; 3 Kyoto Cattle Genetics Section, Beef Information & Genetics Institute, Inc, Otsu, Shiga 520-0865, Japan

## Abstract

**Background:**

Marbling, defined by the amount and the distribution of intramuscular fat and measured as beef marbling score (BMS), is an economically important trait of beef cattle in Japan. We recently reported that a single nucleotide polymorphism (SNP), namely, *c.-312A>G*, in the *endothelial differentiation*, *sphingolipid G-protein-coupled receptor*, *1 *(*EDG1*) gene was associated with the BMS level in the Japanese Black beef cattle population of Oita prefecture, with the *G *allele being associated with a high level of the BMS. Thus, the *c.-312A>G *SNP seems to be a candidate marker for marker-assisted selection. In this study, we investigated whether this association could be replicated in 3 other independent Japanese Black cattle populations and analyzed the effect of the SNP genotypes on the carcass traits other than the BMS.

**Findings:**

Statistically significant differences in the BMS level were detected among the genotypes of the *c.-312A>G *SNP in the Japanese black beef cattle populations of Miyazaki (*P *= 0.0377) and Nagasaki (*P *= 0.0012) prefectures, and marginal difference was detected in the Kagoshima prefecture population (*P *= 0.0786). The *G *allele in the SNP was associated with an increase in the BMS level.

The *G *allele also seemed to have a favorable influence, if any, on the carcass weight, rib eye area and rib thickness of the cattle populations.

**Conclusions:**

These findings suggest that the association of the *c.-312A>G *SNP with the BMS level in the Japanese Black beef cattle population was replicated in other beef cattle populations, and revealed favorable effects of the *G *allele on the beef productivity in the general Japanese Black beef cattle population. Thus, we concluded that the *c.-312A>G *SNP is useful for effective marker-assisted selection to increase the BMS level in Japanese Black beef cattle.

## Background

Intramuscular fat deposition (marbling) measured as beef marbling score (BMS) is one of the economically important traits of beef cattle [1]. A high level of the BMS enriches the taste and tenderness of beef, improving the palatability [2-4], therefore, the BMS affects the evaluation of beef quality [1]. The BMS is regarded as the most important trait especially in Japan. Thus, it would be desirable to construct a more effective marker-assisted breeding scheme for increasing the BMS level in Japanese Black beef cattle.

We recently showed that a single nucleotide polymorphism (SNP), namely, *c.-312A>G*, in the *endothelial differentiation, sphingolipid G-protein-coupled receptor, 1 *(*EDG1*) gene was associated with the BMS level in a Japanese Black beef cattle population in Oita prefecture, with the *G *allele of the SNP being associated with a high level of the BMS [5].

Thus, it was necessary to investigate whether this association could be replicated in other independent populations of Japanese Black beef cattle and to analyze the effects of the SNP genotypes on the carcass traits other than the BMS, in order to confirm the application of the *c.-312A>G *SNP to effective marker-assisted selection.

### Samples and data

We used 3 independent Japanese Black cattle populations, namely, the Kagoshima, Miyazaki and Nagasaki prefecture populations, and studied the association of the *c.-312A>G *SNP with the BMS, subcutaneous fat thickness (SFT), carcass weight (CWT), rib eye area (REA), and rib thickness (RT). For the Kagoshima, Miyazaki and Nagasaki prefecture populations, respectively, 489, 160 and 191 paternal half-sib progeny steers (1 to 110, 1 to 18, and 1 to 36 steers per sire) produced from 72, 45, and 40 sires were used. Adipose tissue specimens of the progeny steers were collected for genotyping the SNP. DNA samples were prepared from the materials using DNeasy Blood & Tissue Kit (QIAGEN, Hilden, Germany).

The BMS, SFT, CWT, REA and RT were measured according to the Japanese meat grading system by certified graders from the Japan Meat Grading Association (Tokyo, Japan) [1]. The predicted breeding values of the BMS, SFT, CWT, REA and RT for the progeny steers were used as the phenotypic values in this study. The breeding values were predicted separately for each of the 3 populations using carcass records of Japanese Black steers and heifers fattened in the Kagoshima, Miyazaki and Nagasaki prefectures.

Data were analyzed by the REML method using the MTDFREML program [6], and the genetic and environmental variances were estimated. The BLUP option in the program using the estimated variance components was chosen to predict the breeding values for animals with a single trait model. The sex, market-year, and farm of the animals were considered as the fixed effects. The fattening period and slaughter age were also considered as up to quadratic covariates. The fattening period denotes the period from the start of the fattening to shipping to the market for each animal. Random effects included additive genetic effects of the individuals; that is, the animal model was adopted to predict the breeding values.

This study conformed to the guidelines for animal experimentation of the Graduate School of Agriculture, Kyoto University (Kyoto, Japan).

### SNP genotyping

The *c.-312A>G *SNP was genotyped by the PCR-restriction fragment length polymorphism method as described previously [5]. Using this method, 378-bp PCR fragments containing the SNP site were amplified and *MscI*-digested into 163- and 215-bp fragments at the *A *allele, but not the *G *allele: the *GG *homozygotes, the *AA *homozygotes and the *AG *heterozygotes yielded 1 band (378 bp), 2 bands (163 and 215 bp) and 3 bands (163, 215, and 378 bp), respectively.

The observed frequencies of the SNP genotypes in the 3 populations are shown in Table 1. The observed and expected heterozygosity values at the SNP conformed to the Hardy-Weinberg equilibrium in all the populations. The frequencies of the *G *allele of *c.-312A>G *SNP in the Kagoshima and Miyazaki prefecture populations were consistent with the frequency of this allele in the Oita prefecture population determined in our previous study [7], whereas the allele frequency in the Nagasaki prefecture population was lower [7].

**Table 1:** Frequencies of the *c.-312A>G *SNP genotypes in the 3 populations

Population	Genotype	No. of animals	Frequency
Kagoshima (n = 489)	*GG*	166	0.339
	*AG*	242	0.495
	*AA*	81	0.166
Miyazaki (n = 160)	*GG*	44	0.275
	*AG*	91	0.569
	*AA*	25	0.156
Nagasaki (n = 191)	*GG*	33	0.173
	*AG*	94	0.492
	*AA*	64	0.335

### Association analysis

Statistically significant differences in the BMS level were detected among the genotypes of the SNP in the Miyazaki and Nagasaki prefecture populations, by analysis using a model that included the SNP genotype as the fixed effect and the sire as the random effect (Table 2). The BMS level in the *GG *homozygotes was significantly higher than that in the *AA *homozygotes, and the values in the heterozygotes were intermediate between those in the 2 homozygotes (Table 2). Theses results were consistent with the data obtained in our previous study in the Oita prefecture population [5]. In the Kagoshima prefecture population, the effects of the SNP genotypes reached marginal significance for the BMS level, with the *GG *homozygotes showing a tendency to exhibit the highest values among the 3 genotypes. These results suggest that the association of the *c.-312A>G *SNP with the BMS level was replicated in the general Japanese Black beef cattle populations.

**Table 2:** Effects of the *c.-312A>G *SNP genotypes on BMS, BFT, CWT, REA and RT in the 3 populations

			Genotype^§^		
			
Trait*	Population	*P*-value	*GG*	*AG*	*AA*
BMS	Kagoshima	0.0786	0.42 ± 0.05	0.28 ± 0.04	0.37 ± 0.07
	Miyazaki	0.0377	0.43 ± 0.13^a^	0.36 ± 0.09^a^	-0.07 ± 0.17^b^
	Nagasaki	0.0012	0.52 ± 0.18^a^	0.01 ± 0.11^b^	-0.32 ± 0.13^b^
SFT	Kagoshima	0.1256	-0.59 ± 0.22	-0.89 ± 0.18	-1.36 ± 0.31
	Miyazaki	0.0619	0.88 ± 0.26	0.45 ± 0.18	-0.14 ± 0.34
	Nagasaki	0.7401	0.05 ± 0.45	0.25 ± 0.27	-0.07 ± 0.32
CWT	Kagoshima	0.1927	8.83 ± 1.60	6.62 ± 1.32	3.83 ± 2.28
	Miyazaki	0.1811	-0.19 ± 0.50	0.25 ± 0.35	-1.12 ± 0.66
	Nagasaki	0.0014	16.30 ± 3.69^a^	5.78 ± 2.19^a^	-0.49 ± 2.65^b^
REA	Kagoshima	0.0029	1.26 ± 0.23^a^	0.45 ± 0.19^b^	-0.02 ± 0.34^b^
	Miyazaki	0.2642	0.71 ± 0.26	0.48 ± 0.18	0.00 ± 0.35
	Nagasaki	0.0960	0.13 ± 0.30	-0.48 ± 0.18	-0.67 ± 0.22
RT	Kagoshima	0.2868	1.14 ± 0.20	0.73 ± 0.16	0.92 ± 0.28
	Miyazaki	0.4833	-0.12 ± 0.42	0.45 ± 0.29	-0.12 ± 0.55
	Nagasaki	0.0002	3.30 ± 0.96^a^	1.50 ± 0.57^b^	-1.32 ± 0.69^b^

*BMS, Beef marbling score; SFT, Subcutaneous fat thickness (mm); CWT, Carcass weight (kg); REA, Rib eye area (cm^2^); RT, Rib thickness (mm).

^§^The breeding values are shown as estimates ± SE.

^a, b^Estimates at different genotypes without a common letter in their superscripts significantly differ (*P *< 0.05).

The effect of the SNP genotype on the BMS level was not statistically significant in the Kagoshima prefecture population (*P *> 0.05). The predominant breeding objective is the BMS in the Miyazaki and Nagasaki prefectures, while it is the CWT in the Kagoshima prefecture (Fujita T, personal communication). Thus, many quantitative trait loci (QTL) for the BMS are thought to be fixed in the Miyazaki and Nagasaki prefecture populations, but not in the Kagoshima prefecture population. Our present study might not have sufficient power to detect the association of the *c.-312A>G *SNP with the BMS level in the Kagoshima prefecture population, because of the larger number of segregating QTL for the BMS in the Kagoshima prefecture population as compared with that in the other two populations. Further study using a larger number of samples is needed for the Kagoshima prefecture population.

The effect of the SNP genotype was not statistically significant for the SFT (Table 2), consistent with the results obtained in the Oita prefecture population [5]. In addition, the SNP genotype had no significant effect on the CWT in the Kagoshima and Miyazaki prefecture populations, on the REA in the Miyazaki and Nagasaki prefecture populations, and on the RT in Kagoshima and Miyazaki prefecture populations (Table 2). However, the effect of the SNP genotype reached statistical significance for the CWT in the Nagasaki prefecture population, for the REA in the Kagoshima prefecture population, and for the RT in the Nagasaki prefecture population. In these cases, the CWT, REA and RT values were significantly higher in the *GG *homozygotes than in the *AA *homozygotes, and the values in the heterozygotes were intermediate between those in the 2 homozygotes. Thus, just like its effect on the BMS, the effects of the *G *allele on carcass traits other than the BMS, if any, seemed to be favorable rather than deleterious.

In this study, we demonstrated replication of the association of the *c.-312A>G *SNP with the BMS level, and revealed a favorable effect of the *G *allele on the beef productivity in general Japanese Black beef cattle populations. Together with the results of our previous study [5], these findings suggest that the *c.-312A>G *SNP in *EDG1 *is useful for effective marker-assisted selection to increase the level of marbling in Japanese Black beef cattle.

## Competing interests

The authors declare that they have no competing interests.

## Authors' contributions

SS carried out the genotyping and statistical analyses and drafted the manuscript. TM carried out further statistical analyses. YT, HM and FM participated in the sample and data collection. TY and YS participated in the design and coordination of the study and helped draft the manuscript. All authors read and approved the final manuscript.
